# Exploring the Anti-inflammatory Effects of Protopine Total Alkaloids of *Macleaya Cordata* (Willd.) R. Br.

**DOI:** 10.3389/fvets.2022.935201

**Published:** 2022-07-05

**Authors:** Zhen Dong, Yu-hong Wang, Zhao-shan Tang, Chang-hong Li, Tao Jiang, Zi-hui Yang, Jian-guo Zeng

**Affiliations:** ^1^College of Veterinary Medicine, Hunan Agricultural University, Changsha, China; ^2^Key Laboratory of Chinese Veterinary Medicine in Hunan Province, Hunan Agricultural University, Changsha, China; ^3^State Key Laboratory of Chinese Medicine Powder and Innovative Drugs, Hunan University of Chinese Medicine, Changsha, China; ^4^Hunan MICOLTA Biological Resources Co., Ltd, Changsha, China

**Keywords:** *Macleaya cordata* (Willd.) R. Br., protopine total alkaloids, acute inflammation, anti-inflammatory, network pharmacology, molecular docking

## Abstract

*Macleaya cordata* (Willd). R. Br. is a Chinese medicinal plant commonly used externally to treat inflammatory-related diseases such as arthritis, sores, and carbuncles. This study aimed to evaluate the anti-inflammatory activity of protopine total alkaloids (MPTAs) in *Macleaya cordata* (Willd.) R. Br. *in vivo* tests in rats with acute inflammation showed that MPTA (2.54 and 5.08 mg/kg) showed significant anti-inflammatory activity 6 h after carrageenan injection. Similarly, MPTA (3.67 and 7.33 mg/kg) showed significant anti-inflammatory activity in the mouse ear swelling test. In addition, the potential mechanisms of the anti-inflammatory effects of MPTA were explored based on network pharmacology and molecular docking. The two main active components of MPTA, protopine and allocryptopine, were identified, and the potential targets and signaling pathways of MPTA's anti-inflammatory effects were initially revealed using tools and databases (such as SwissTargetPrediction, GeneCards, and STRING) combined with molecular docking results. This study provides the basis for the application of MPTA as an anti-inflammatory agent.

## Introduction

*Macleaya cordata* (Willd.) R. Br. (*M. cordata*) is a plant of the opium poppy family that is widely distributed in China and, to a lesser extent, in Japan (1). According to the Tang dynasty Chinese herbal text, *M. cordata* is highly toxic and should not be taken internally but should be used externally to treat arthritis, carbuncles, analgesia, and swelling (2). Modern research has shown that the main pharmacologically active substances in *M. cordata* are alkaloids, which are also responsible for their toxicity (3–6). Modern pharmacological studies have shown that the alkaloids of *M. cordata* have antibacterial, antifungal, antitumor, anti-inflammatory, and snail-killing properties (7–11). They are widely used in animal husbandry to improve the intestinal health of animals, enhance the body's immunity, and improve production performance (12–14). However, most current research revolves around benzo[c]phenanthridine alkaloids, represented by sanguinarine and chelerythrine, and less research has been done on protopine alkaloids.

Inflammation is the basis for many physiological and pathological processes and is an adaptive response with clinical manifestations defined as “redness, swelling, heat, and pain,” the classical causes of infection and tissue damage (15). Acute inflammation (AI) triggered by the classical pathway involves coordinated transport of plasma and leukocytes; mobilization of mast cells and macrophages; production of chemokines, cytokines, and arachidonic acid; local tissue liquefaction to prevent microbial transfer; the killing of microorganisms; and tissue repair (16). Suppose the acute response to inflammation fails to subside or steps in the inflammatory process are blocked. In that case, the inflammation persists and develops into a chronic inflammation characterized by granulomas and tertiary lymphoid structures, leading to DNA oxidation and tumor development (17). Inflammation (especially chronic inflammation) is a complex event, and in some specific infectious diseases, inflammation harms the organism far beyond the pathogen itself, such as the SARS-CoV-2-induced inflammatory storm (18, 19). Inflammation is also an important player in all types of disease, and controlling it is sometimes an optimal option when the causative factors are unknown (17, 20, 21). In clinical practice, the use of non-steroidal anti-inflammatory drugs (NSAIDs) is the primary means of controlling the inappropriate occurrence of inflammation, but with the widespread use of NSAIDs comes concerns about their safety, particularly the potential cardiovascular and gastrointestinal bleeding risks, and despite the announced withdrawal of valdecoxib and rofecoxib, the development of anti-inflammatory drugs has not ceased (22, 23). As research has progressed, many compounds of plant origin have shown good anti-inflammatory activity and safety and are gradually becoming a key source for drug development. *M. cordata* alkaloids exhibit good anti-inflammatory activity, but the protopine alkaloids' anti-inflammatory activity and mechanism of action are not yet known.

Network pharmacology is a new approach based on systems biology, integrating pharmacology, computer science, and other multidisciplinary approaches that play an important role in drug discovery (24). It can explore the mode of action of drugs from multiple perspectives, better reflecting the systematic and holistic view of drug action and providing an effective paradigm for converting ethnopharmacology from empirical to evidence-based research, accelerating the process of modernizing traditional ethnic groups medicines (25).

In this study, the anti-inflammatory activity of protopine total alkaloid (MPTA) was tested using the classic animal models of acute inflammation, the rat paw edema model, and the mouse ear swelling model, and the available information was mined by combining network pharmacology and molecular docking techniques to screen potential key action targets and signaling pathways. The data generated in this study will guide and support further in-depth research on the anti-inflammatory effects of MPTA and drug development.

## Materials and Methods

### Materials

**Figure 1:**
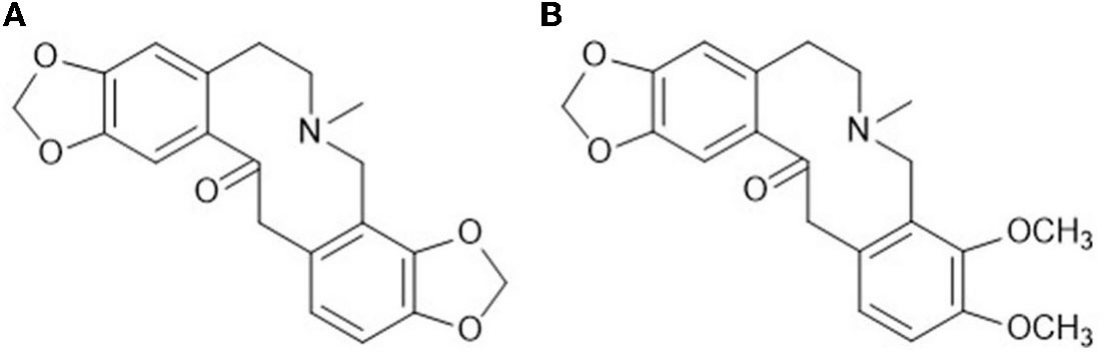
Chemical structure formulas of the main components. **(A)** Protopine (PRO); **(B)** allocryptopine (ALL).

Protopine total alkaloids [protopine alkaloids ≥50%, of which protopine (PRO) ≥35%, allocryptopine (ALL) ≥15%, all other low content components have been characterized (26)] were supplied by Hunan MICOLTA Biological Resources Co., Ltd. (Liuyang, China), Lot 170501, and samples are stored in State Key Laboratory of Chinese Medicine Powder and Innovative Drugs. Protopine, sanguinariumchloride, and chelerythrine were provided by the National Institutes for Food and Drug Control (Beijing, China). Allocryptopine was supplied by Hunan MICOLTA Biological Resources Co., Ltd. (Liuyang, China). Chromatography-grade acetonitrile was purchased from Sinopharm Chemical Reagent Co., Ltd. (Shanghai, China). Ultrapure water was produced in-house by the laboratory (Milli-Q A10, Boston, MA, USA). The rest of the reagents were analytically pure. The structural formulas of the compounds are shown in Figure 1. Prednisone acetate was purchased from Xianju Pharma Co., Ltd. (Taizhou, China). Xylenes were purchased from Hunan Hui Hong Reagent Co., Ltd. (Changsha, China). Carrageenan was purchased from Beijing Solarbio Science and Technology Co., Ltd. (Beijing, China). The YLS-7B paw volume detector was provided by Xuzhou Lihua Electronic Technology Development Co., Ltd. (Xuzhou, China). An 8-mm ear punch was provided by GENE and I Technology Co., Ltd. (Beijing, China). A model AY-120 electronic analytical balance was provided by SHIMAZU (China) Co., Ltd. (Shanghai, China).

### Characteristic Profiling of MPTA

The method of characteristic profiling has been further optimized and built on methods already available in the laboratory (27). The LC method uses a Shimadzu Model 20A HPLC equipped with an SPD-20A detector and SIL-10A autosampler. The separation was performed on an Agilent HC-C18 (250 × 4.6 mm, 5 μm) column at 35°C with a mobile phase of A (acetonitrile): B (0.1% phosphoric acid), flow rate: 0.8 ml/min, injection volume: 5 μl, gradient. The elution procedure was as follows: 0–14 min, the gradient held 25% A; 14–27 min, the gradient from 25 to 60% A; 27–29 min, the gradient from 60 to 25% A; and 29–35 min, held 25% A.

A total of nine different batches of MPTA were taken and tested according to the above LC method. The similarity of each batch to this common pattern was calculated by fitting the common pattern (R) with the full spectrum from 0 to 35 min using the software of the Chromatographic Fingerprint Evaluation System for Chinese Medicine (National Pharmacopeia Commission 2004). The results of the methodological validation are not shown in this study.

### Animals

SPF male ICR mice weighing 18–22 g [Certificate of Compliance No.: 43004700043051, License No.: SCXK (Xiang) 2014-0011] were purchased from HUNAN SJA LABORATORY ANIMAL CO., LTD. Male and female SPF SD rats weighing 130–150 g [Certificate of Compliance No.: 43004700043026, License No.: SCXK (Xiang) 2014-0011] were purchased from Hunan Sja Laboratory Animal Co., Ltd. All experimental animals were housed under GLP laboratory conditions and acclimatized for 5 days before testing, with free access to food and water. All animal tests were approved by the Animal Ethics Committee of the Hunan University of Chinese Medicine.

### *In vivo* Anti-inflammatory Activity

#### Paw Edema Induced by Carrageenan

The test for carrageenan-induced paw edema was performed according to the description in the literature, with minor modifications (28). A total of 50 rats were randomly divided into a control group (pure water, g/ml), a positive control group (prednisone acetate, PA, 10.8 mg/kg) and three test groups of 10 rats each at different doses of MPTA (1.27, 2.54, and 5.08 mg/kg). The volume of the drug administered by gavage was 10 ml/kg once daily for 7 days. Before the last dose, the volume under the right ankle joint of the rats was measured using a paw volumetric instrument as the pre-inflammatory volume. After 30 min of the last dose, the rats were injected subcutaneously with 0.1 ml of 1% carrageenan into the right hind paw to cause inflammation. The right subankle volume was measured at 0.5, 1, 2, 4, and 6 h post-inflammation. The paw edema degree and inhibition rate were calculated according to the following formulas. Paw edema degree = postinflammatory paw volume – preinflammatory paw volume, and paw edema inhibition rate = [(control paw edema – paw edema in drug administration group) / control paw edema] × 100%.

#### Xylene-Induced Auricular Edema

The xylene auricular edema test is based on literature protocols (29). A total of 50 mice were randomly divided into a control group (pure water, g/ml), a positive control group (PA, 7.8 mg/kg), and three test groups of MPTA at different doses (1.83, 3.67, and 7.33 mg/kg), with 10 mice in each group. The volume of the drug administered by gavage was 20 ml/kg once daily for 7 days. After 60 min of the last dose, 30 μl of xylene was evenly applied to both the anterior and posterior sides of the right ear of the mice, and the mice were dislocated from the cervical vertebrae after 4 h of inflammation. Auricular edema degree = right ear piece weight – left ear piece weight. Auricular edema inhibition rate = [(control auricular edema – administered auricular edema) / control auricular edema] × 100%.

### Network Pharmacology and Molecular Docking

#### Screening of Active Ingredients

Gastrointestinal absorption and drug-like properties were screened for two major compounds in MPTA (PRO and ALL) using the SwissADME tool (http://www.swissadme.ch/) (30), and the screening criteria were high gastrointestinal absorption and bioavailability score ≥ 0.55 (31). Compounds having >10% bioavailability in rats can be given a bioavailability score (ABS). Most cationic and amphoteric compounds meeting the rule of five achieve ABSs of 0.55 and 0.17, respectively (32, 33). Canonical SMILES of compounds are available in PubChem (https://pubchem.ncbi.nlm.nih.gov/) (34, 35).

#### Target Prediction and Disease Target Acquisition

Prediction of potential targets of active ingredients was performed using the SwissTargetPrediction (http://www.swisstargetprediction.ch/) (36). Targets for acute inflammation are available in the GeneCards database (https://www.genecards.org/) (37). Gene symbols were obtained and checked in the UniProt database (http://www.UniProt.org/) for the species condition *Homo sapiens* (38).

#### Protein Interaction Analysis and Network Visualization

The predicted targets of the compounds were intersected with the inflammatory targets, and the intersected target genes were imported into the STRING database (https://cn.string-db.org/) for protein–protein interaction (PPI) network analysis. Species for *Homo sapiens* and isolated nodes were excluded and evaluated according to the degree values of the nodes (39). Construction of “drug-compound-target-disease” networks and PPI networks using the Cytoscape 3.7.1 software. Screening of core targets was performed using the CytoHubba plugin's MCC algorithm (40).

#### GO and KEGG Pathway Enrichment Analysis

The core targets were entered into the DAVID platform (https://david.ncifcrf.gov), with the species set to “Homo sapiens,” and the main biological processes and metabolic pathways were analyzed and enriched (41). The top 10 data with *p* < 0.05 under each category were selected for visualization. A “compound-target-pathway” network was constructed using the Cytoscape 3.7.1 software.

### Molecular Docking

The structures of the target proteins and compounds were downloaded from the RCSB PDB (https://www.rcsb.org) and PubChem (https://pubchem.ncbi.nlm.nih.gov/) databases, respectively (42). The storage formats of the compound structures were transformed by Openbabel software. Solvent and organic molecules were removed using PyMOL software. Molecular docking was performed with AutoDock software. The AutoGrid and AutoDock modules were used to enable semiflexible docking and obtain affinities for small molecule compounds and protein receptors.

### Statistical Analysis of Data

Data from this experiment are expressed as the mean ± SD and were statistically analyzed using SPSS 26.0 statistical software. The measurement data were first subjected to ANOVA with the chi-square test. Data with *p* > 0.05 were statistically analyzed by the LSD (L) method, and those with *p* < 0.05 were statistically analyzed by Dunnett's method.

## Results

### Characteristic Profiling of MPTA

**Figure 2:**
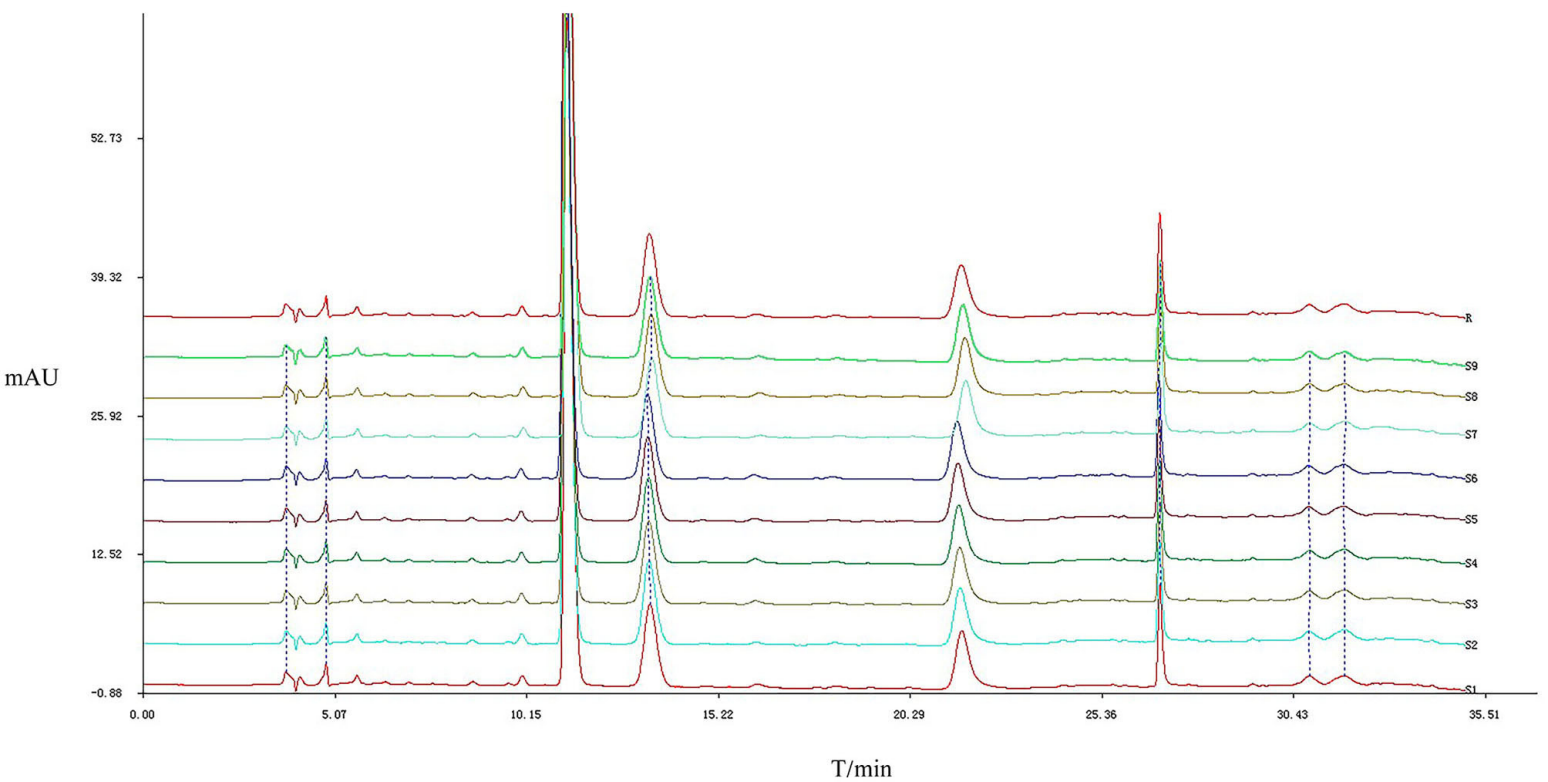
Characteristic chromatograms of different batches of MPTA. The horizontal coordinate is the retention time. The vertical coordinate is the signal value.

**Figure 3:**
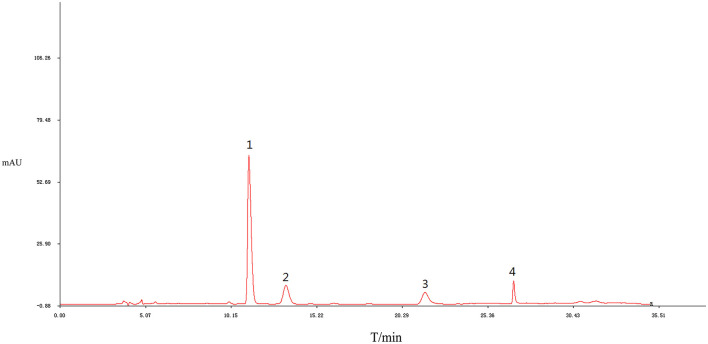
Control characteristics spectrum of MPTA. Peak 1: protopine; Peak 2: allocryptopine; Peak 3: sanguinarine; and Peak 4: chelerythrine.

The results of the similarity calculations are shown in Table 1. Based on the relevant parameters of the HPLC profiles obtained for the nine batches of the test drug Figure 2, all chromatographic separations from MPTA can be eluted within 35 min. Using a half-peak width of 20, a slope of 1,000, and a minimum peak height of 1.5% of the highest peak for integration, a comparison of the chromatograms of the batches revealed that a total of four distinct peaks were common to all batches, all four peaks could be confirmed by known controls, and the system-generated control characteristic profiles are shown in Figure 3.

**Table 1:** Similarity results between different batches of MPTA and shared patterns (*n* = 9).

**Batch number**	**S1**	**S2**	**S3**	**S4**	**S5**	**S6**	**S7**	**S8**	**S9**
Similarity	0.992	0.992	0.992	0.992	0.995	0.995	0.995	0.995	0.995

*The similarity of the shared pattern (R) is specified as 1*.

### *In vivo* Anti-inflammatory Activity

#### Paw Edema Induced by Carrageenan

As shown in Table 2, compared with the control group, the swelling of the paw carrageenan in the PA group was significantly lower at 0.5, 1, 2, 4, and 6 h after inflammation, and the difference was statistically significant (*p* < 0.05). The swelling of the paws of rats in the MPTA 5.08 mg/kg group was significantly reduced at 6 h after the inflammation, and the difference was statistically significant (*p* < 0.05), while the swelling of the paws of rats in the MPTA 5.08 mg/kg group was reduced at 0.5, 1, 2, and 4 h after the inflammation, but the difference was not statistically significant (*p* > 0.05). In the MPTA 2.54 mg/kg group, the swelling of rats' paws was significantly reduced at 6 h after the inflammation caused by MPTA 2.54 mg/kg. The difference was statistically significant (*p* < 0.05). In contrast, the swelling of rats' paws was reduced at other time points after the inflammation caused by MPTA 2.54 mg/kg, but the difference was not statistically significant (*p* > 0.05). The swelling of the rats in the MPTA 1.27 mg/kg group was reduced at all time points, but the difference was not significant (*p* > 0.05).

**Table 2:** Effect of MPTA on paw edema in rats (*n* = 10).

**Groups**	**Preinflammatory volume (mL)**	**0.5 h Post-inflammatory**		**1 h Post-inflammatory**		**2 h Post-inflammatory**		**4 h Post-inflammatory**		**6 h Post-inflammatory**	
		**Edema degree (mL)**	**Inhibition rate (%)**	**Edema degree (mL)**	**Inhibition rate (%)**	**Edema degree (mL)**	**Inhibition rate (%)**	**Edema degree (mL)**	**Inhibition rate (%)**	**Edema degree (mL)**	**Inhibition rate (%)**
Control	0.999 ± 0.109	0.281 ± 0.089	—	0.338 ± 0.126	—	0.369 ± 0.143	—	0.303 ± 0.101	—	0.269 ± 0.124	—
PA	0.992 ± 0.084	0.199 ± 0.085*	29.2	0.217 ± 0.107**	35.8	0.181 ± 0.085*	50.9	0.119 ± 0.057**	60.7	0.049 ± 0.044**	81.7
MPTA 5.08 mg/kg	0.975 ± 0.068	0.233 ± 0.093	17.1	0.268 ± 0.075	20.7	0.253 ± 0.061	31.4	0.184 ± 0.060	39.2	0.096 ± 0.050*	64.3
MPTA 2.54 mg/kg	0.967 ± 0.081	0.252 ± 0.060	10.3	0.298 ± 0.082	11.8	0.281 ± 0.115	23.8	0.195 ± 0.063	35.6	0.104 ± 0.082*	61.3
MPTA 1.27 mg/kg	0.988 ± 0.099	0.260 ± 0.089	7.8	0.306 ± 0.077	9.5	0.291 ± 0.155	21.1	0.223 ± 0.111	26.4	0.140 ± 0.089	50.0

Comparison with the control group ^*^P <0.05, ^**^P <0.01.

##### Xylene-Induced Auricular Edema

The results are shown in Table 3. Compared with the control group, the right ear edema was significantly reduced in the PA group of mice after xylene inflammation, and the difference was statistically significant (*p* < 0.05); the right ear weight and ear edema were significantly reduced in the MPTA high- and medium-dose groups and the difference was statistically significant (*p* < 0.05); the right ear weight and edema were reduced in the MPTA low-dose group of mice, and there was a certain inhibitory effect, but the difference was not statistically significant (*p* > 0.05).

**Table 3:** Effect of MPTA on auricular edema in mice (*n* = 10).

**Groups**	**Left ear weight (mg)**	**Right ear weight (mg)**	**Edema degree (mg)**	**Inhibition rate (%)**
Control	15.8 ± 1.4	22.2 ± 1.4	6.4 ± 1.4	—
PA	15.4 ± 1.7	18.9 ± 2.2*	3.5 ± 1.0**	45.3
MPTA 7.33 mg/kg	15.3 ± 1.4	19.2 ± 2.4*	3.9 ± 1.8*	35.9
MPTA 3.67 mg/kg	15.4 ± 1.1	19.8 ± 1.9*	4.4 ± 1.3*	31.2
MPTA 1.83 mg/kg	15.5 ± 1.5	21.7 ± 2.2	6.2 ± 2.4	7.8

Comparison with the control group ^*^P <0.05, ^**^P <0.01.

### Network Pharmacology and Molecular Docking

#### Determination of Active Ingredients

The two main active ingredients (PRO and ALL) were screened for drug properties. As seen in Table 4, PRO and ALL have good oral availability and drug-like properties.

**Table 4:** Pharmaceutical properties of active ingredients.

**Component**	**GI absorption**	**Drug-likeness**					
		**Lipinski**	**Ghose**	**Veber**	**Egan**	**Muegge**	**Bioavailability score**
PRO	High	Yes	Yes	Yes	Yes	Yes	0.55
ALL	High	Yes	Yes	Yes	Yes	Yes	0.55

#### Target Prediction and Disease Target Acquisition

**Figure 4:**
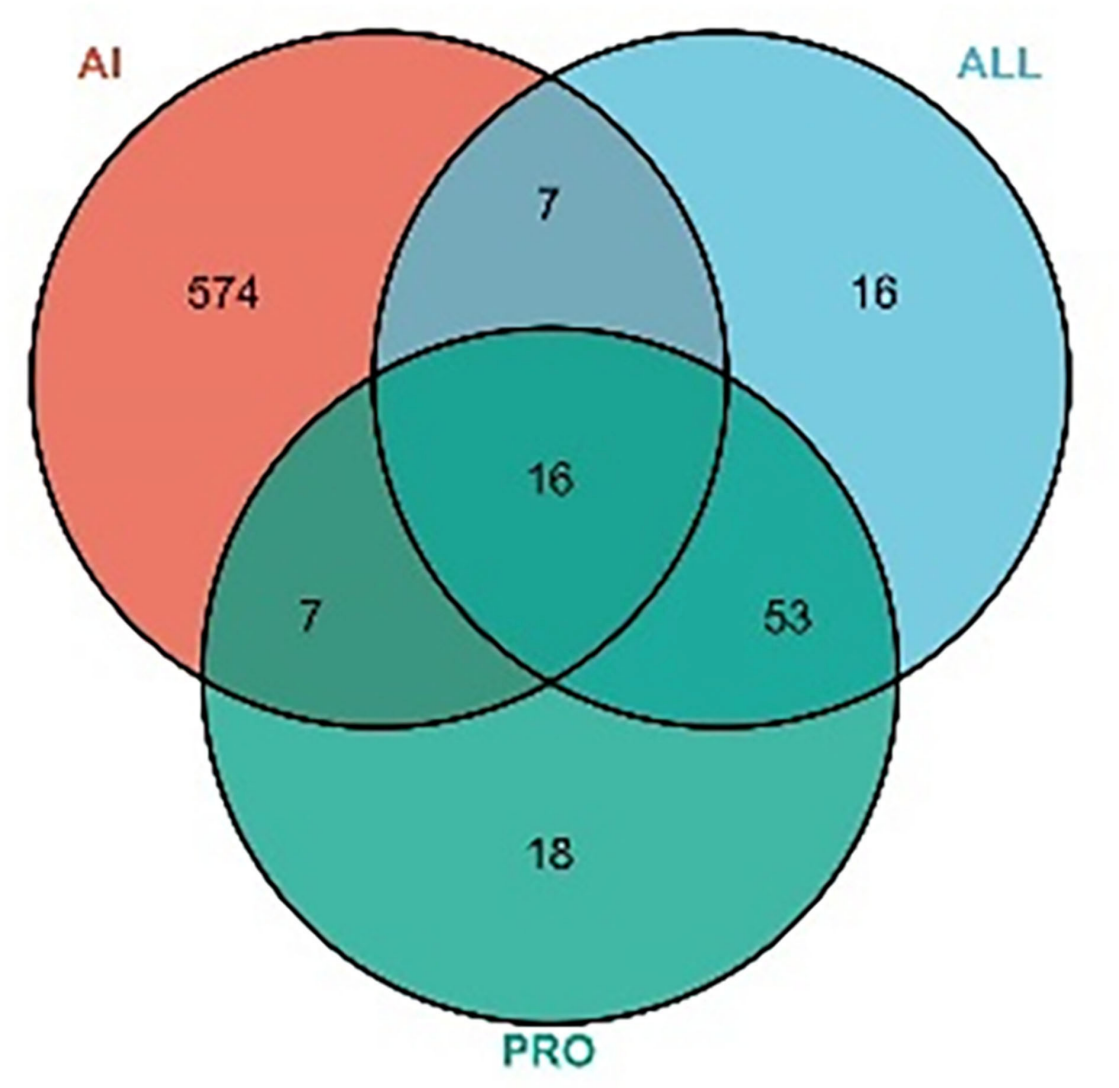
VENN diagram for MPTA and AI. Number of potential targets of MPTA against acute inflammation (AI). ALL, number of potential targets predicted for allocryptopine; PRO, number of potential targets predicted for protopine; AI, number of target genes for acute inflammation screened from the disease database.

A total of 117 targets were obtained by predicting PRO and ALL. A total of 8,633 AI-associated targets were obtained from the GeneCards database, and 604 targets with correlation scores >10 were screened. A total of 30 intersecting targets were associated with AI for PRO and ALL, as shown in Figure 4.

#### PPI Analysis and Network Visualization

**Figure 5:**
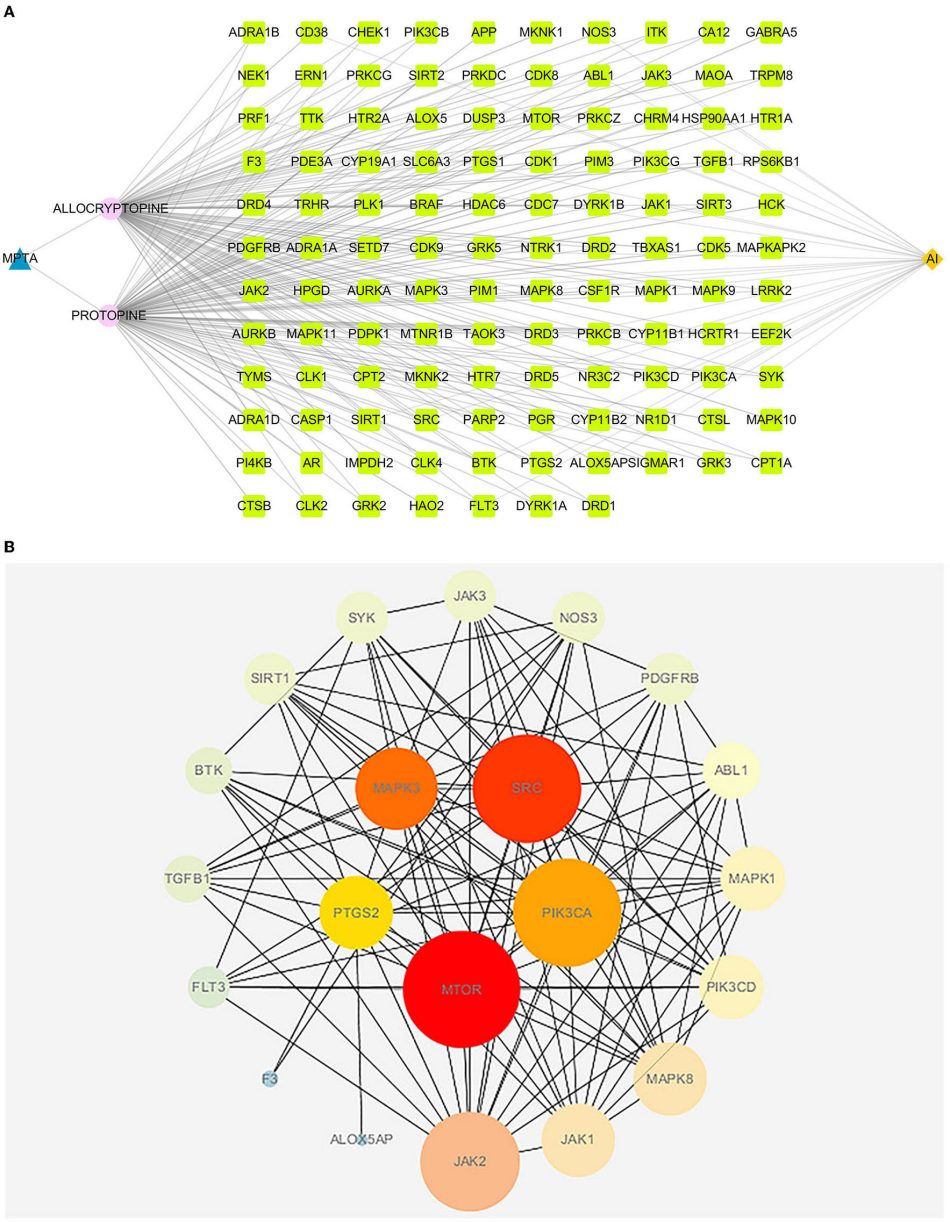
**(A)** The “drug-component-disease-target” network diagram of MPTA's anti-inflammatory effects. Blue triangular nodes represent MPTA; pink circular nodes represent active compounds; dark yellow diamond nodes represent acute inflammation (AI); and bright green square nodes represent targets of MPTA for inflammation. **(B)** PPI diagram of MPTA's anti-inflammatory targets. The circular nodes represent the potential target proteins of MPTA's anti-inflammatory action; the larger the radius of the nodes, the greater their degree; the lines between the nodes represent the interaction between the targets; the darker the color of the lines, the stronger the interaction between the nodes. The five darkest nodes in the center are the core targets calculated by the Hubba algorithm.

The active ingredient, AI and intersecting targets in MPTA were constructed into a “drug-component-target-disease” network diagram by Cytoscape 3.7.1 software (Figure 5A), and the intersecting targets were imported into the STRING database for protein interaction analysis, and the results were imported into Cytoscape 3.7. The results were imported into the Cytoscape 3.7.1 software to map the PPI network (Figure 5B), and then the top five core targets in the network (MTOR, SRC, MAPK3, PIK3Cam, and PTGS2) were obtained using the MCC algorithm in the Cytohubba plugin.

#### GO and KEGG Enrichment Analysis

**Figure 6:**
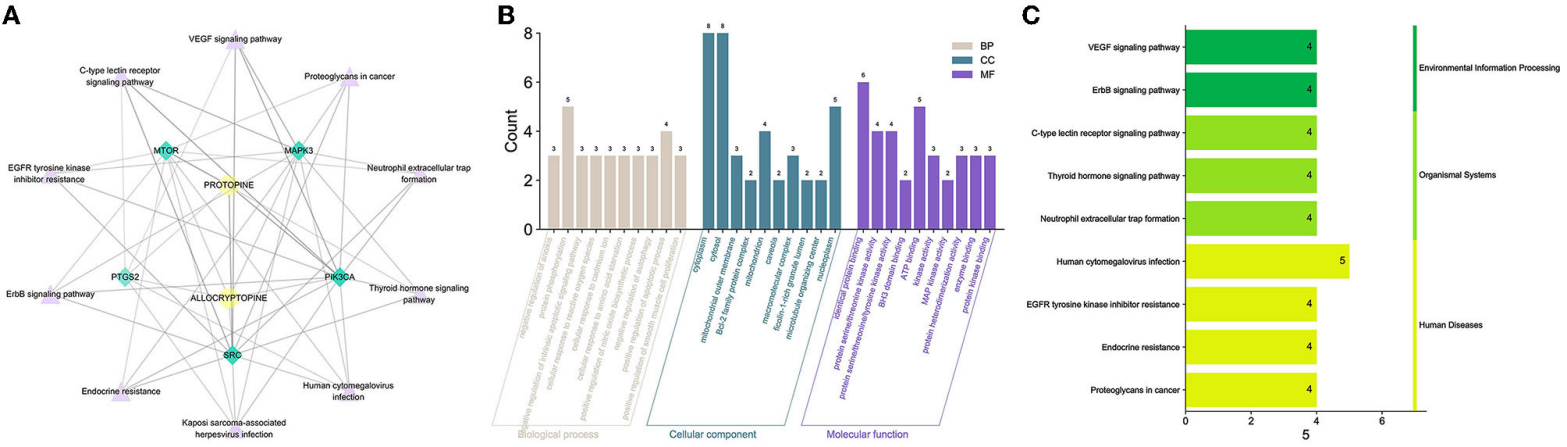
**(A)** The “drug-signaling pathway-target” network of MPTA's anti-inflammatory effects. Yellow circular nodes represent active compounds; green diamond nodes represent core targets screened; and lavender triangular nodes represent signal transduction pathways. **(B)** GO enrichment analysis of MPTA. The three colors represent biological processes, cellular components, and molecular functions in that order. The horizontal coordinate represents the biological function to which the core target is enriched; the vertical coordinate represents the number of genes. **(C)** KEGG analysis of MPTA. The horizontal coordinate represents the number of genes involved in the enrichment; the vertical coordinate represents the KEGG pathway that was enriched.

The core protein targets were enriched by the DAVID database for GO biology and KEGG signaling pathways. A total of 81 anti-inflammatory signaling pathways were enriched by MPTA (*p* < 0.05), and the top 10 pathways were selected according to the significance of the *p*-value. The “drug-signaling pathway-target” network was constructed using the Cytoscape 3.7.1 software (Figure 6A). GO enrichment revealed that MPTA was involved in 49 biological processes (BP), four cellular components (CC), and seven molecular functions (MF) (*p* < 0.05). Figure 6B shows the top 10 entries (<10 for CC and MF) in the three classifications under MPTA-related bioenrichment, including biological processes such as positive regulation of smooth muscle cell proliferation, platelet activation, phosphorylation, and the Fc-gamma receptor signaling pathway involved in phagocytosis; proteins are mainly in the cellular environment, such as the cytoplasm and plasma membrane; molecular functions such as protein serine/threonine kinase activity, ATP binding, and phosphoprotein binding. Figure 6C shows the top 10 signaling pathways, which are mainly involved in three types of biometabolic pathways, i.e., environmental information processing, organismal systems, and human diseases, including Kaposi's sarcoma-associated herpesvirus infection, human cytomegalovirus infection, signaling pathways of vascular endothelial growth factor, ErbB signaling pathway, and C-type lectin receptor signaling pathway.

#### Molecular Docking

**Figure 7:**
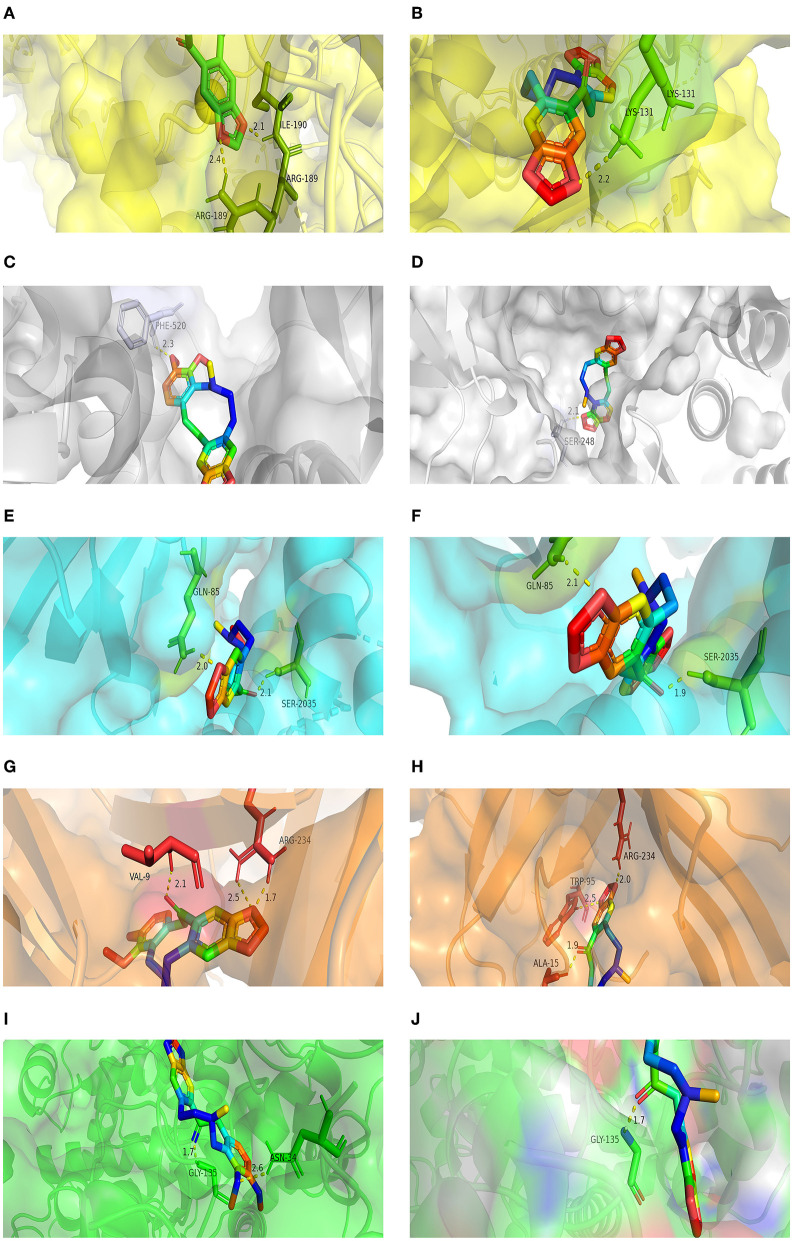
Molecular docking results for MPTA. **(A)** Docking mode for ALL-MAPK3; **(B)** docking mode for PRO-MAPK3; **(C)** docking mode for ALL-SRC; **(D)** docking mode for PRO-SRC; **(E)** docking mode for ALL-MTOR; **(F)** docking mode for PRO-MTOR; **(G)** docking mode for ALL-PIK3CA; **(H)** docking mode for PRO-PIK3CA; **(I)** docking mode for ALL-PTGS2; and **(J)** docking mode for PRO-PTGS2.

The molecular docking of PRO and ALL to the five core targets (MTOR, SRC, MAPK3, PIK3CA, and PTGS2) in the PPI network was considered good binding with binding energies ≤ −5 kcal/mol and hydrogen bonding numbers ≥ 1. The binding energies of the two active ingredients docked to the core targets are shown in Table 5, which indicates that both active ingredients have good binding properties to all five core targets. The results showed that both active ingredients had good binding properties to the five core targets. The well-bound molecular docking diagrams are shown in Figure 7.

**Table 5:** Binding energy of the active ingredients in MPTA to the core targets.

**Compounds**	**Affinity**									
	**MAPK3**		**SRC**		**MTOR**		**PIK3CA**		**PTGS2**	
	**Combination of energy (kcal/mol)**	**Number of hydrogen bonds**	**Combination of energy (kcal/mol)**	**Number of hydrogen bonds**	**Combination of energy (kcal/mol)**	**Number of hydrogen bonds**	**Combination of energy (kcal/mol)**	**Number of hydrogen bonds**	**Combination of energy (kcal/mol)**	**Number of hydrogen bonds**
ALL	−6.34	1	−6.87	1	−7.46	2	−7.42	2	−7.24	1
PRO	−7.25	1	−7.31	1	−7.66	2	−7.86	2	−7.43	1

## Discussion

Inflammation is an important and complex defense mechanism. Inflammation is a double-edged sword; when regulated, the inflammatory response is beneficial to the organism, and while uncontrolled, it can be harmful. It is important to manage acute inflammation in which the causes and processes are relatively clear and more manageable and in which the disease becomes more complex and difficult to control if it progresses to chronic inflammation (43, 44).

Carrageenan-induced paw edema and xylene-induced auricular edema models are two most commonly used *in vivo* models of acute inflammation and are often used to test the anti-inflammatory activity of drugs (45, 46). The inflammation induced by carrageenan is mainly characterized by edema at the injection site, with the greatest swelling occurring 4–5 h after injection (28). In addition, carrageenan-induced inflammation has a typical biphasic phenomenon and essentially consists of three phases (28, 47). The early phase within 1–2 h after injection consists of a 5-hydroxytryptamine-mediated primary phase and a secondary kinin-mediated secondary phase, during which blood flow and vascular permeability at the injection site increase and can be used to assess the activity of histamine and bradykinin receptor antagonists (48–50). The late phase 2 h after injection is due to the body producing large amounts of prostaglandins, particularly prostaglandin E. This process involves the release of arachidonic acid (AA) from the cell membrane *via* cPLA2, which is then converted to PGH2 *via* COX, and PGH2 is further catalyzed prostaglandins by tissue-specific prostaglandin synthase (51). Through the nonspecific inhibition of COX-1/2, nonsteroidal anti-inflammatory drugs (NSAIDs) exert their anti-inflammatory and analgesic activity (52). The edema that occurs in the preinflammatory phase gradually intensifies to the peak of inflammation within 3 h, after which the degree of edema slowly diminishes, so it is reasonable that the edema model constructed in this study peaks at 3 h (53). The results showed that MPTA at 2.54 and 5.08 mg/kg achieved the highest inhibition rate of edema at 6 h and showed some dose dependence (1.27–2.54 mg/kg). Based on the study results, it is speculated that the anti-inflammatory activity of MPTA may occur through influencing the activity of COX, similar to NSAIDs. The results of MPTA inhibition of xylene-induced auricular edema showed consistency with carrageenan-induced toe swelling, with results showing that 3.67 and 7.33 mg/kg of MPTA significantly reduced the degree of swelling.

Based on the anti-inflammatory activity results, this study combined network pharmacology and molecular docking techniques to preliminarily investigate the potential mechanisms of action by which MPTA exerts its anti-inflammatory effects. Further mining of the active ingredients using web tools, such as SwissADME, SwissTargetPrediction, STRING, DAVID, and public databases, resulted in the prediction of 117 potential targets for MPTA and 8,633 AI-related targets. Intersection analysis of related targets yielded 30 common targets for MPTA and AI, such as MAPK1, MTOR, JAK2, and NOS3. In PPI network analysis, the MCC algorithm is considered to be the best method for obtaining key genes (54). The MCC algorithm obtained five core targets, namely, MTOR, SRC, MAPK3, PIK3CA, and PTGS2, which play an important role in the PPI network. MTOR is a class of serine/threonine kinases whose stability affects the expression of cytokines in T cells, participates in the regulation of cell growth, apoptosis and autophagy, and has an important regulatory role in inflammation (55, 56). SRC can be activated following the involvement of immune response receptors and cytokine receptors, and inhibiting SRC can significantly improve neutrophil inflammation (57). Both MAPK3 and PIK3CA play important roles in signaling cascades that mediate various cellular immune-related signaling pathways, such as the MAPK and NF-kappa B pathways (58, 59). PIK3CA plays a regulatory role in cell phagocytosis and is closely associated with COX-2 expression (60, 61). PTGS2, or COX-2 (inducible), is an important rate-limiting enzyme in the prostaglandin biosynthetic pathway with a specific role in the inflammatory response and is one of the important targets of action of NSAIDs (62).

The GO enrichment analysis showed that the biological functions of the core targets are enriched in the cytoplasm and membranes and have multiple roles (e.g., inphosphorylation and platelet activation). The VEGF signaling pathway – enriched by KEGG – involves lymphangiogenesis, and VEGF1 can be expressed on the membranes of macrophages, promotes cytokine/chemokine production, stimulates inflammatory responses, and is often used as a biomarker for microinflammation (63, 64). The ErbB signaling pathway promotes apoptosis in inflammatory macrophages, maintains epithelial cell survival, and plays an important role in suppressing intestinal inflammation (65, 66). The C-type lectin receptor pathway plays an important role in immune regulation, as it controls the functional differentiation of T cells and triggers cascade signals during bacterial and fungal infections (67–69). In addition, macrophage-induced C-type lectin (Mincle) induces persistent tissue damage inflammation (aseptic inflammation) due to cell death clearance (70). Thyroid hormones are important for maintaining normal metabolism in the body, and their signaling pathway is involved in the metabolism of lipids and carbohydrates (sugars). It has been shown that the glycolytic pathway can regulate the activity of inflammatory vesicles in macrophages, leading to increased sepsis and that inhibiting this process can reduce the damage caused by microbial sepsis (71, 72). The molecular docking results showed that both active compounds in MPTA bound well to the five core protein targets, suggesting that these high-affinity targets may be key in the anti-inflammatory action of MPTA, with possible interactions with PTGS2 also explaining the results seen in *in vivo* anti-inflammatory assays.

PRO is often used as one of the quality markers (Q-markers) of *Corydalis yanhusuo* W.T. Wang plays an important role in exerting blood-activating and analgesic effects (73, 74). Due to its good pharmacological properties, it has been studied as a potential anti-neurodegenerative disease and antithrombotic agent. Several studies have shown that PRO can inhibit acetylcholinesterase activity and promote abnormal tau protein degradation in Alzheimer's disease (75–77). In addition, there are some reports on the anti-inflammatory properties of PRO. An isoquinoline alkaloid extract of *Hypecoum erectum* containing PRO exhibited anti-inflammatory activity comparable to dexamethasone in a carrageenan-reduced rat model (78). PRO also attenuates and prevents LPS-induced inflammatory damage *via* TRL4 and MAPK/NF-kappa B (79, 80). Few studies have examined ALL, although some have reported its potential antiarrhythmic activity (81–83). Only one study reported the protective effect of ALL against oxidative stress-induced neuronal damage (84). It is well-known that the persistence of oxidative stress induces inflammation and further mediates the development of acute and chronic inflammation in multiple systems, such as obesity, asthma, cardiovascular disease, diabetes, and neuroinflammation (85–90). It is generally accepted that antioxidants are beneficial for controlling inflammation (91). In particular, analgesia is often an important consideration in developing anti-inflammatory drugs. PRO and ALL have been found to modulate dopamine receptors and sodium and potassium channels to exert analgesic effects (92–94). Therefore, ALL and PRO have good potential for development as anti-inflammatory agents.

## Conclusion

The results from animal models of acute inflammation support the anti-inflammatory activity of MPTA, and network pharmacology and molecular docking results suggest that MPTA may exert its anti-inflammatory effects by acting on targets such as MTOR, SRC, MAPK3, PIK3CA, and PTGS2 and through signaling pathways, such as VEGF, ErbB, and the C-type lectin receptor pathway.

## Data Availability Statement

The original contributions presented in the study are included in the article/supplementary material, further inquiries can be directed to the corresponding authors.

## Ethics Statement

The animal study was reviewed and approved by Animal Ethics Committee of Hunan University of Chinese Medicine.

## Author Contributions

ZD and J-gZ: conceptualization. ZD and Y-hW: methodology. ZD: software, data curation, writing—original draft preparation, writing—review and editing, and visualization. ZD and TJ: validation. ZD and Z-hY: formal analysis. C-hL and J-gZ: resources. C-hL and Z-sT: supervision. Z-hY: project administration. All authors have read and agreed to the published version of the manuscript.

## Conflict of Interest

Z-sT and C-hL were employed by the Hunan MICOLTA Biological Resources Co., Ltd. The remaining authors declare that the research was conducted in the absence of any commercial or financial relationships that could be construed as a potential conflict of interest.

## Publisher's Note

All claims expressed in this article are solely those of the authors and do not necessarily represent those of their affiliated organizations, or those of the publisher, the editors and the reviewers. Any product that may be evaluated in this article, or claim that may be made by its manufacturer, is not guaranteed or endorsed by the publisher.
